# Investigation of the Antibacterial and Antibiofilm Activity of Selenium Nanoparticles against *Vibrio cholerae* as a Potent Therapeutics

**DOI:** 10.1155/2022/3432235

**Published:** 2022-03-23

**Authors:** Sareh Bagheri-Josheghani, Bita Bakhshi

**Affiliations:** Department of Bacteriology, Faculty of Medical Sciences, Tarbiat Modares University, Tehran, Iran

## Abstract

*Vibrio cholerae* is a major cause of severe diarrhea, which is ecologically flexible, and remains as a major cause of death, especially in developing countries. Consecutive emergence of antibiotic-resistant strains is considered to be as one of the major concerns of the World Health Organization (WHO). Nanoparticles as a new nonantibiotic therapeutic strategy have been widely used in recent years to treat bacterial infections. The present study aimed to investigate the antibacterial and antibiofilm effect of selenium nanoparticles (SeNPs) in vitro against *V. cholerae* O1 ATCC 14035 strain. SeNPs were prepared and characterized using ultraviolet-visible (UV-Vis) spectroscopy, DLS (dynamic light scattering), zeta potential measurement, and Fourier transform infrared (FTIR) analysis. The concentration of SeNPs was calculated by ICP (inductively coupled plasma) method. Also, 3-(4,5-dimethylthiazol-2-yl)-2,5-diphenyl-2H-tetrazolium bromide (MTT) assay was employed to assess the cytotoxic effect of SeNPs on Caco-2 cells. Antibacterial and antibiofilm activity of SeNPs was determined by broth microdilution and crystal violet assays, respectively. The average particle size of SeNPs was 71.1 nm with zeta potential −32.2 mV. The SEM images supported the uniform spherical morphology of the prepared nanoparticles. The antibiofilm effect of SeNPs was evident at concentrations of 50–200 *μ*g/mL. This study results provided evidence that SeNPs are safe as an antibacterial and antibiofilm agent against *V. cholerae* O1 ATCC 14035 strain.

## 1. Introduction

Recently, nanotechnology is designed and applied in the pharmaceutical industry. Nanomaterials have extensive applications in the field of biotechnology, medicine, and chemistry due to their small particle size, targeted effects, fewer side effects, solubility, and pharmacokinetics; their biodistribution is also easy to administer, offering a market advantage to their developers [1].

Cholera epidemics are considered as an important public health concern in developing countries. So far, *Vibrio cholerae* has caused seven cholera pandemics in the world. According to the World Health Organization (WHO), 1.3 to 4.0 million new cases of cholera are diagnosed each year, resulting in 21000 to 143000 deaths per year worldwide [2]. Antibiotic treatment is an adjunct therapy used for acute diarrhea in cholera patients to reduce the duration and severity of diarrhea; it is also a beneficial measure that could be taken to control cholera epidemics. However, WHO does not recommend the indiscriminate use of antibiotics because overuse of antibiotics contributes to the emergence of antimicrobial resistance and multiple antibiotic resistance (MAR). This antimicrobial resistance makes bacterial infections more difficult to treat and increases fatality rates during cholera outbreaks. However, many countries have reported the emergence of toxigenic *V. cholerae* strains resistant to frequently used antimicrobial agents [3].

Therefore, the development of new strategies to combat *V. cholerae* infections is a necessity, and the solution may lie in nanotechnology [4]. Among nanoparticles, selenium nanoparticles (SeNPs) are considered as nontoxic and bioactive agents with low toxicity and high biocompatibility [5]. Therefore, SeNPs are described as nanomaterials that could be used for therapeutic purposes. Besides, SeNPs as a strong antibacterial agent have been authenticated to inhibit bacterial growth [6]. Biofilms are complex communities of surface-bound bacteria that are embedded together in a self-produced matrix. Since these communities of bacteria are difficult to treat, there is a need for novel antibiofilm inhibitors [7].

Antibacterial activity of SeNPs against multiple bacterial species had been reported, including *Escherichia coli* [6–8], *Staphylococcus aureus* [8, 9], *Pseudomonas aeruginosa* [10], *Streptococcus mutans* [9], *Enterococcus faecalis* [9, 11], *Candida albicans* [11], and *S*. *pyogenes* [12]. Evidences suggest that *V. cholerae* could produce biofilm-like aggregates through intestinal infection, which could play a critical role in bacterial pathogenesis. The antibiofilm potential of SeNPs has also been investigated in various studies against different bacterial species, including *S. aureus*, *P. aeruginosa*, and *Proteus mirabilis* [13]; *Bacillus cereus*, *E*. *faecalis*, *S. aureus*, *E*. *coli* O157 : H7, *Salmonella Typhimurium*, and *S*. *enteritidis* [14]; and a number of clinical strains of bacteria [15].

However, previous studies have not specifically evaluated the potential antimicrobial and antibiofilm activity of SeNPs against *V. cholerae* strains.

By relying on these results, the present study was performed to investigate the antimicrobial and antibiofilm effect of SeNPs on *V. cholerae* strains. Therefore, SeNPs were first synthesized using a chemical reduction approach, then their antibacterial and antibiofilm activity against *V. cholerae* O1 ATCC 14035 strain was evaluated. The cytotoxic potential of SeNPs was evaluated against Caco-2 cell line. Herein, the antimicrobial and antibiofilm activity of SeNPs against *V. cholerae* O1 ATCC 14035 was reported for the first time. All of the findings strongly verified the potential antimicrobial and antibiofilm effect of SeNPs as a novel therapeutic agent against *V. cholerae* O1 ATCC 14035.

## 2. Materials and Methods

### 2.1. Ethical Considerations

The research was appraised and approved by the Research Ethics Committee of Tarbiat Modares University under code number: IR.MODARES.REC.1399.059 approval ID before it began.

### 2.2. Bacterial Strains

*V. cholerae* O1 ATCC 14035 strain used in this study was obtained from the archive of Tarbiat Modares University of Medical Sciences (Tehran, Iran).

### 2.3. Synthesis of Selenium Nanoparticles (SeNPs)

SeNPs was synthesized using reducing sodium selenite in the presence of ascorbic acid according to a study by Vahdati slightly modified. Synthesis of SeNPs by this method has biocompatibility and good reducing properties [9, 16]. Briefly, 58.13 mM ascorbic acid (Merck, Germany) was added to Na_2,_SeO_3_ (5H_2_O) at a concentration of 1.2 mM (Merck, Germany). Ascorbic acid was introduced into the resulting solution at a 4 : 1 ratio of ascorbic acid/Na_2_SeO_3_.

Ascorbic acid was added dropwise while stirring at 1300 rpm at room temperature. The formation of SeNPs was visible with a color change in the solution from white to orange. The solution was centrifuged at 12000 rpm and then pellet washed. Pellet was resuspended in 1 mL of sterile double-distilled water. In addition, Tween 20 (30 *μ*L/20 mL) was used to prevent the aggregate of SeNPs during the synthesis process.

### 2.4. Characterization of SeNPs

The prepared SeNPs identity was verified using an ultraviolet-visible (UV-Vis) spectrophotometer (Perkin-Elmer, Thermo Scientific, USA) in the 200–500 nm wavelength range. The size distribution and zeta potential of the prepared SeNPs were specified using a Zeta Sizer Nano Series (Zetasizer Nano ZS, Malvern, Worcestershire, UK) after sonicating for 10 min in a bath-type sonicator.

Using the Inductively Coupled Plasma-Atomic Absorption Spectroscopy (ICP-AAS) technique, the amount of selenium in the prepared nanoparticles was determined. The concentration of SeNPs was determined according to the standard selenium concentration curve. Selenium standards were made from sodium selenite salt at concentrations of 0–100 ppm.

Acid digestion of nanoparticles was carried out using a solution of 2% nitric acid. Selenium standards were then prepared from sodium selenite salt at concentrations of 1–100 ppm. In order to identify the major structural groups in SeNPs, Fourier transform infrared (FTIR) analysis was performed using a FTIR spectrometer (FTIR, PerkinElmer, USA) in the wavenumber range of 400–4000 cm^−1^.

In addition, both scanning electron microscopy (FE-SEM, Tescan Mira3) and transmission electron microscopy (TEM, Phillips EM 2085) (100 kV) methods were employed to study and analyze the morphology of the prepared nanoparticles. All reported images were estimated by ImageJ software.

### 2.5. Sterility Test

In this study to investigation of sterility of prepared nanoparticles, SeNPs were cultured on the following media, including thioglycolate media, nutrient agar, blood agar, MacConkey agar, and Sabouraud dextrose agar, and then placed under anaerobic and aerobic conditions. The optimal culture period was also examined [17].

### 2.6. Cell Culture and Assessment of Cytotoxicity of SeNPs

To investigate the cytotoxic potential of SeNPs, human colon carcinoma (Caco-2) cell line was prepared (IBRC, Iran, Tehran), and the optimal dose of SeNPs was calculated by examining mitochondrial dehydrogenases activity using 3-(4,5-dimethylthiazol-2-yl)-2,5-diphenyl-2H-tetrazolium bromide (MTT) assay kit (Yekta Tajhiz, Iran). Briefly, Caco-2 cells were cultured and grown in monolayers using DMEM culture medium (Dulbecco's Modified Eagle Medium) complemented with 10% fetal bovine serum (FBS), 50 IU/mL penicillin, and 50 *μ*g/mL streptomycin. Afterwards, the grown cells were incubated at 37°C for 24 h using a 5% CO_2_ incubator. Caco-2 cells were then removed by trypsinization. To obtain the best results, the cells were implanted in a 96-well plate with approximately 10^6^ cells per well in their exponential growth phase and inoculated with different concentrations (0–200 *μ*g/mL) of SeNPs for 24, 48, and 72 h after incubation in CO_2_ incubator. The supernatant of Caco-2 cells without stimuli was used as negative control. Each test was performed along with a control containing complete medium with no cells as blank; nanoparticles and MTT reagent without cells were used as blanks. Following exposure to the composite, 100 *μ*L of reconstituted MTT was added to each well and incubated again for 4 h. Finally, detergent reagent with a volume equal to the volume of the original culture medium (usually 100 *μ*L) was added up and down by a pipet to completely dissolve the resulting MTT formazan crystals. After thorough mixing, the resulting solution optical density was read immediately in a microplate reader using a microplate spectrophotometer (Epoch, USA) at the background absorbance of multiwell plates at 690 nm and subtract at 570 nm. All assays were carried out in triplicate. Cell viability was represented and compared to the controls. The viability of controls (without stimuli) was set at 100%, and all values were represented as percentage. Respective IC50 values (minimum particle concentration causing 50% cell death) were determined by GraphPad Prism Software Version 8 by employing regression analysis.

### 2.7. Antibacterial Activity Evaluation Using Microbroth Dilution Method

Antibacterial activity of SeNPs was examined through microdilution susceptibility testing according to the Clinical and Laboratory Standards Institute (CLSI) guidelines against *V. cholerae* ATCC 14035 strain. Briefly, about 0.1 mL of serial dilutions of 12.5, 25, 50, 100, and 200 mg/mL of SeNPs were transferred into a 96-well plate. *V. cholerae* strains were cultured in Mueller-Hinton broth (MHB) culture medium at 37°C for 24 h and grown to log phase to reach an optical density (OD) of 1 : 0 (10^8^ CFU/mL). After 24 h, 5 × 10^5^ CFU/mL (0.01 mL) of bacteria (*V. cholerae* O1 ATCC 14035) and 0.1 mL of MHB were added to each well and incubated overnight at 37°C for 24 h. Each sample was then serially diluted to obtain a dilution of 10^−5^. Culture medium containing bacteria without SeNPs was prepared as positive control, and culture medium without bacteria was prepared as negative control. To count the colonies, 10 *μ*L of each sample was cultured onto Mueller-Hinton agar (MHA) and incubated at 37°C for 24 h. After incubation, CFU of bacterial cells was enumerated based on the following formula:

number of colonies × 100 × inverse dilution factor (M07, CLSI, 2019).

All experiments were performed with three replicates.

### 2.8. Antibiofilm Assay

The antibiofilm activity of SeNPs was assessed in this study using crystal violet method. Briefly, *V. cholerae* O1 ATCC 14035 strain was cultured in 1 mL of BHI (brain-heart infusion) broth medium (Merck, Germany) at 37°C for 24 h to obtain an optical density (OD) of 1 : 0 (10^8^ CFU/mL). Each well of a 96-well plate was filled with 0.1 mL of BHI broth supplemented with 0.5% (w/v) sucrose. The wells were then inoculated with 0.1 mL of SeNPs (concentrations used were 0–200 mg/mL) and 0.01 mL of *V. cholerae* O1 ATCC 14035 suspension (10^8^ CFU/mL) and incubated at 37°C for 24 h. The medium of each well was gently taken away, and the wells were rinsed three times with 0.2 mL of phosphate-buffered saline (PBS PH7.2) to eliminate free-floating bacterial strains. Attachment of *V. cholerae* O1 ATCC 14035 to the 96-well plate was evaluated by staining with 1% (wt/vol) crystal violet solution. The 96-well plate was again washed to remove excess stain and kept for drying. Finally, the biofilm mass was destained using 95% ethanol for 45 min, and a microplate spectrophotometer (Epoch, USA) was used to measure the OD value of biofilm formation related to crystal violet at 570 nm. The optical density (OD) values of different samples with SeNPs were compared with the control sample. The OD value was considered as biofilm formation on the surface of the 96-well plate. The test was performed in triplicate [18].

### 2.9. Statistical Analysis

To assess the significant differences, GraphPad Prism Software Ver. 8 was used by employing one-way ANOVA test. A *P* value of less than 0.05 was considered as significant. The obtained results were represented as the mean ± standard deviation (SD). All experiments were performed in triplicate.

## 3. Results

### 3.1. Characterization of SeNPs

**Figure 1:**
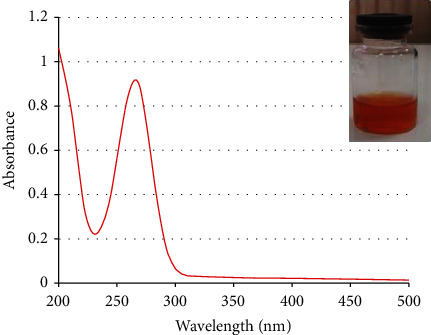
UV-vis spectrum of SeNPs.

The appearance of a sharp peak at around 266 nm displayed by UV-Vis absorption spectra is assigned to SeNPs. The characterization of SeNPs absorption peak is shown in Figure 1.

**Figure 2:**
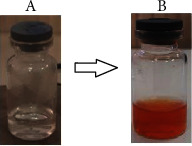
The color change from white (a) to orange (b) shows a decrease in selenium to elemental selenium (Se^0^) and the formation of Se nanoparticles.

Also, the color change from white to orange shows a decrease in selenium to elemental selenium (Se^0^) and the formation of Se nanoparticles shown in Figure 2.

**Figure 3:**
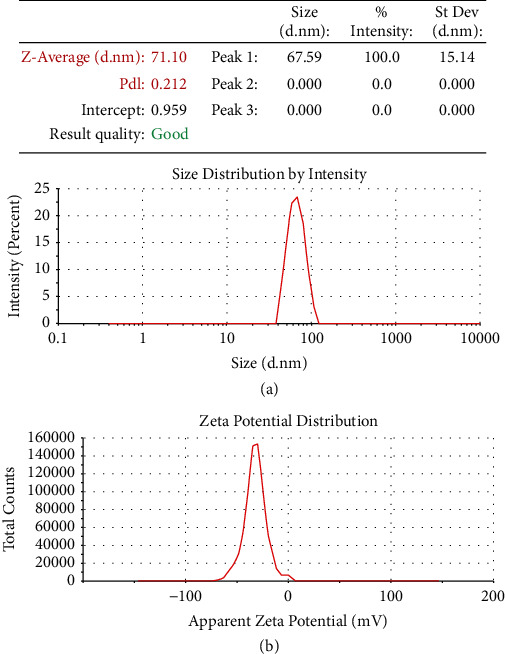
(a) DLS analysis of the synthesized SeNPs and (b) zeta potential of the synthesized SeNPs.

According to the DLS (dynamic light scattering) analysis results, SeNPs zeta potential was determined to be about −32.2 mV. The average size of the synthesized SeNPs was determined to be around 71.1 ± 10.3 nm. The polydispersity index (PDI) in DLS analysis was 0.21, confirming the homogeneous and nondispersive size of SeNPs (Figure 3).

**Figure 4:**
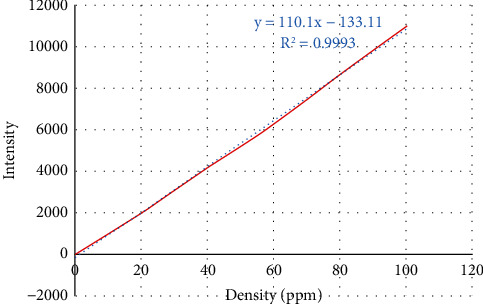
Calibration curve of concentration of standard selenium solutions.

Using ICP method, the calibration curves were plotted for selenium standards, and selenium content in nanoparticles was determined to be 0.654 *μ*g/ml (Figure 4).

**Figure 5:**
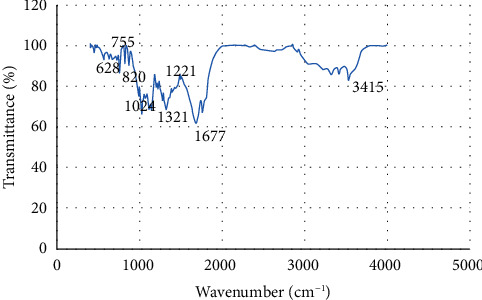
FTIR analysis of selenium nanoparticles (SeNPs).

Furthermore, Se-OH groups and nanoparticle surface hydroxyl groups also peak in the range of 400–4000 cm^−1^. The surface groups of Se-C have an absorption peak in the range of 1000–1800 cm^−1^ due to the presence of ascorbic acid in the synthesis of these particles. These functional groups confirm the contribution of different reducing and stabilizing agents in the synthesis of SeNPs [19–21]. FTIR spectra of SeNPs are shown in Figure 5.

The absorption 1221.62 cm^−1^ represents that the complexation takes place between C-N or -C-N group and selenium ions [21]. The line at 1321 cm^−1^ after the synthesis of SeNPs was attributed to C-H band vibrations, or syringyl ring breathing with C=O stretching. The presence of a peak at 758 cm^−1^ was assigned to the ring-vibrating modes of ortho-substituted aromatics [22]. Bands detected at 820, 1024, and 1321 are related with C-O stretch [20, 23]. The band at 1677  cm^−1^ might represent the asymmetric and symmetric stretching vibrations of carboxylates (some carboxylic acids as products of deep ascorbic acid oxidation adsorbed on SeNPs) [24]. In the FTIR spectrum of SeNP, the peaks in the range 3200–3526 cm^−1^ and 820 cm^−1^ signifying correspond to the diverse hydroxyl groups (-OH). A peak at 3415 cm^−1^ is assigned to O-H stretching vibration of alcohol and phenol groups. The peak at 3513.38 cm^−1^ corresponds to the bending vibration of O-H. The bands at 628 cm^−1^ correspond to the stretching and bending vibrations of Se-O, which may be attributed to the binding of SeNPs to the carbonyl groups from the yields of oxidation of ascorbic acid [24, 25].

**Figure 6:**
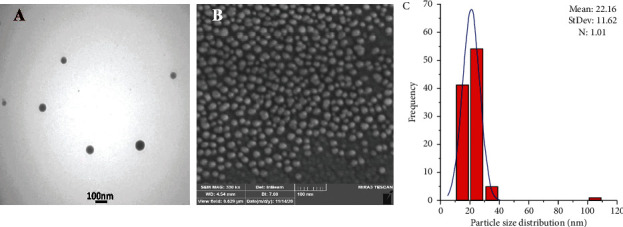
Morphology and size distribution of SeNPs: (a) TEM images of SeNPs, (b) SEM images of SeNPs, and (c) histogram of particle size distribution and the fit curve related to the particle in SEM by ImageJ.

The morphology and size distribution of selenium nanoparticles were investigated using FE-SEM and TEM, showing approximately a spherical and regular shape with an average size of 22.6 nm, the results of which are presented in Figure 6.

### 3.2. Sterility Test

In this test, SeNPs were cultured on the thioglycolate media, nutrient agar, blood agar, and MacConkey agar and then placed under anaerobic and aerobic conditions. Examination of bacterial cultures after 24 and 48 h showed no bacterial growth in the media, revealing the sterility of the synthetized SeNPs.

### 3.3. Cytotoxic Effect of SeNPs

**Figure 7:**
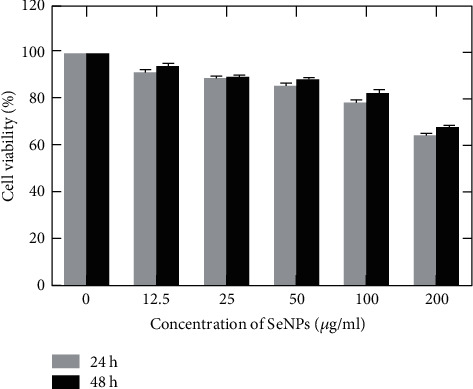
Viability of Caco-2 cells in the presence of different concentrations of SeNPs according to the OD values of 690 nm and subtract at 570 nm after 24 and 48 h.

In this study, the cytotoxic potential of SeNPs (after sterilization) was evaluated against Caco-2 cells using MTT test; the results were determinate as the percentage of cell viability in the presence of different concentrations of SeNPs (Figure 7). The viability of controls (without stimuli) was set at 100%. According to the results, more than 50% of the cells treated with 0–200 ppm of SeNPs for 24 and 48 h were viable. Moreover, the cell viability in the presence of 100 *μ*g/mL of SeNPs after 24 and 48 h was 83.1 and 78.8%, respectively.

### 3.4. Antimicrobial Activity Evaluation of Different Concentrations of SeNPs against *V. cholerae* O1 ATCC 14035 Strain by CFU Counting

**Figure 8:**
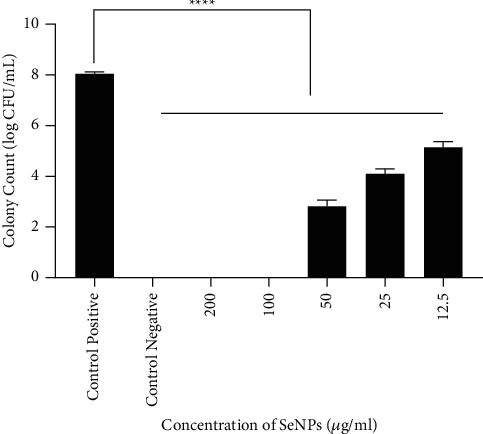
SeNPs showed antimicrobial activity against *V. cholerae* O1 ATCC 14035 strain. Bacterial growth was assessed by CFU counting. Bars are presented as the mean results of three tests ± SD. ^*∗∗∗∗*^*P* value <0.0001 for different concentrations vs. control. Values showed the mean results of three tests ± SD. Data were analyzed by ANOVA test and Bonferroni.

**Figure 9:**
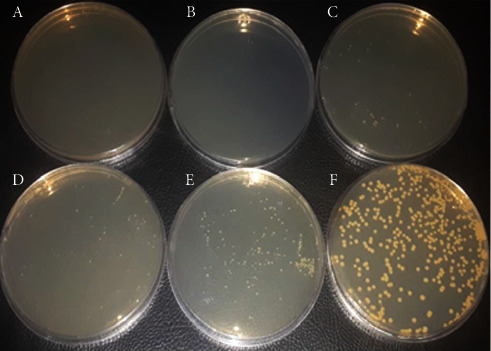
Antibacterial efficacy of SeNP on Mueller-Hinton agar (MHA) medium with different concentrations: (a) 200 *μ*g/mL; (b) 100 *μ*g/mL; (c) 50 *μ*g/mL; (d) 25 *μ*g/mL; (e) 12.5 *μ*g/mL; and (f) control positive.

As shown in Figures 8 and 9, SeNPs significantly inhibited bacterial growth. The antibacterial effect of different concentrations of SeNPs was evaluated separately. According to the results, the antibacterial effect of SeNPs was more evident at higher concentrations. In fact, the bacterial growth was inhibited in the presence of SeNPs at concentrations higher than 25 *μ*g/mL. The bacterial growth was completely inhibited in cells treated with 200 and 100 *μ*g/mL of SeNPs. Bacterial growth was reduced in the presence of SeNPs at concentrations of 50 and 25 *μ*g/mL. According to the results, the antibacterial activity of SeNPs was significant at concentrations of 200, 100, 50, 25, and 12.5 *μ*g/mL (^*∗∗∗∗*^*P* value <0.0001) compared to the positive control.

### 3.5. Antibiofilm Assay

**Figure 10:**
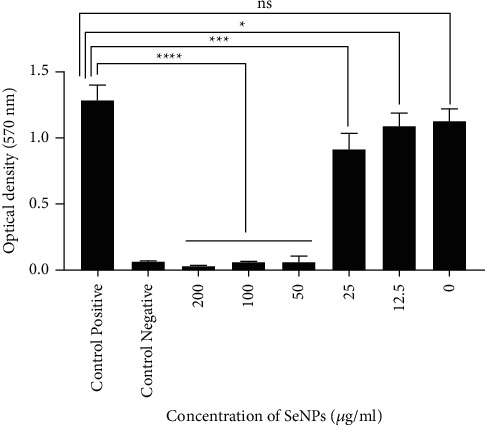
The inhibitory effects of different concentrations of SeNPs on biofilm formation of *V. cholerae* O1 ATCC 14035 strain. Bacterial suspension was incubated with each concentration. ^*∗∗∗∗*^*P* <  0.0001 for concentrations of 200, 100, and 50 of SeNPs vs. positive control; ^*∗∗∗*^*P* for a concentration of 25 of SeNPs vs. positive control, and ^*∗*^*P* value <0.05 for a concentration of 12.5 of SeNPs vs. positive control.

The antibiofilm activity of SeNPs was evaluated against *V. cholerae* O1 ATCC 14035 strain using crystal violet assay, the results of which are presented in Table 1 and Figure 10.

**Figure 11:**
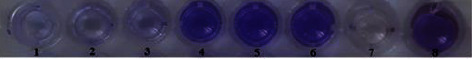
Antibiofilm effect of different concentrations of SeNPs on biofilm formation of *V. cholerae O1* ATCC 14035 strain: different concentrations of SeNPs (1) 200, (2) 100, (3) 50, (4) 25, (5) 12.5, (6) 0 *μ*g/ml, (7) negative control, and (8) positive control.

The antibiofilm activity of SeNPs was higher at concentrations of 200, 100, and 50 *μ*g/mL (^*∗∗∗∗*^*P* value <0.0001) compared to the concentrations of 25 (^*∗∗∗*^*P* value <0.001) and 12.5 *μ*g/mL (^*∗*^*P* value <0.05). Biofilm formation was determined as nonadherent at concentrations of 200 and 100, as weak at a concentration of 50, and as strong at concentrations of 25, 12.5, and 0; however, biofilm formation in no group was determined as intermediate. The results exhibited that SeNPs had inhibitory activity against biofilm formation at concentrations higher than 50 *μ*g/ml. Biofilm formation was measured photometrically at OD = 570 nm, the results of which are presented in Figure 11.

## 4. Discussion

The present study aimed to investigate the antimicrobial and antibiofilm potential of SeNPs against *V. cholerae* in vitro. Recent research has shown that *V. cholerae* could transfer resistance genes as part of mobile genetic elements to other intestinal pathogens. In recent years, the emergence of MDR *V. cholerae* strains has been increasing worldwide, which is considered as a global public health problem. As a result, the need for alternative nonantibiotic approaches to treat *V. cholerae* infections is felt more than ever.

The mechanism of the cytotoxicity of Se NPs has remained indistinct, principally on Caco-2 cells.

The cytotoxic effect of different concentrations (50–200 *μ*g/mL) of SeNPs was tested against Caco-2 cell for 24 and 48 h using MTT test. The results demonstrated that more than 50% of the cells were viable. The present study results are in line with the findings of another study by Raahati et al. and Soflaei et al. [16, 26], reporting the nontoxic nature of SeNPs (100 *μ*g/mL). In other study, the concentration 41.5 ± 0.9 *μ*g/mL of SeNPs had 50% cell death (IC50) in the cells treated [27] . This result is in agreement with a previous study that shows Se NPs exhibit a mild cytotoxic activity on Caco-2 cells [28]. Indumathy et al. reported that cell viability of the SeNPs against HepG2 cell line was with 77%, 63%, and 33.7% of at 2 *μ*g/ ml, 4 *μ*g/ml, and 30 *μ*g/ml concentration, respectively [29].

Previous studies have suggested that nanoparticles could be used as antibacterial agents against *V. cholerae* [30–32]. This study results suggest that SeNPs could be considered as an effective antibacterial and antibiofilm agent and utilized as an efficient approach against *V. cholerae* infections. SeNPs were synthesized in this study by reducing sodium selenite in the presence of ascorbic acid. SeNPs were produced with a diameter of around 71.1 ± 10.3 nm. The present study results showed that SeNPs (12.5–200 *μ*g/mL) have potential antibacterial and antibiofilm activity against *V. cholerae* O1 ATCC strain as suggested by CFU counting. Similarly, another study by Nguyen et al. investigated antibacterial effect of SeNPs to inhibit the growth of food-borne pathogens, *E. coli* O157 : H7, *S. aureus*, *Salmonella*, and *Listeria monocytogenes*. The results demonstrated that SeNPs (spherical in shape with an average diameter of **∼**79 nm) could be potentially employed as an antibacterial agent for inhibiting *S. aureus* growth as well as for food safety applications at the concentration 20–50 *μ*g/mL [28].

In a research carried out by Guisbiers et al., antibacterial activity of SeNPs was assessed against *E. coli* and *S. aureus* isolates. Their study results showed that SeNPs significantly diminished the count of *E. coli* and *S. aureus* strains after 4, 8, and 24 h [8]. The mechanism of antibacterial action of SeNPs is unknown. Research has shown that SeNPs could increase the lag time and substantially reduce the growth rate of *S. aureus* through depleting glutathione (GSH) [33].

In a study by Zhang et al., SeNPs exhibited inhibitory activity against bacterial growth, and the mortality rate of Gram-negative bacteria was much better than that of other bacteria. SeNPs caused the leakage of proteins and polysaccharides after reacting with bio-SeNPs by altering membrane permeability and disrupting bacterial cell walls. Also, changes in the intensity of reactive oxygen species (ROS) by SeNPs could induce antibacterial effects [34].

The results of antibacterial ability of some studies were shown in Table 2.

The results of different studies vary in antibacterial effects of SeNPs on bacteria. The difference between the findings of different studies is mainly due to the difference in the size of nanoparticles and the type of bacteria used. One of the most important factors affecting the antimicrobial properties of nanoparticles is the particle size and concentration. It was considered that smaller nanoparticles had increased the production of ROS than larger surface area to volume ratio inside or out of the cells [38].

In this study with the increase of SeNPs concentration (12.5 to 200 *μ*g/mL), the growth of *V. cholerae* gradually reduced (Figure 8). The results significantly indicated that SeNPs have potential antibacterial activity against *V. cholerae* O1 ATCC and could be employed as an adjunctive antibacterial treatment for *V. cholerae* infections.

New strategies other than conventional antibiotic treatments are needed to control biofilm formation in bacterial infections. *V. cholerae* biofilms have been shown to be hyperinfective; these strains could remain in the environment and increase antibiotic resistance in *V. cholerae* strains. Mature biofilm formation requires the production of matrix proteins, especially RbmA, RbmC, and Bap1. These proteins maintain the structural integrity of the wild-type biofilm [39].

Various studies have shown the effect of different nanoparticles as biofilm inhibitors against different bacteria. The antibiofilm activity of magnesium oxide against *Streptococcus mutans* and *Streptococcus sobrinus* (500 *μ*g/mL) was indicated by Noori and Kareen. They reported that the average nanoparticle is approximately 20.8 nm. [40].

The synthesized AgNPs (the spherical shape, at the size of 55 nM) were used as an inhibitor for controlling biofilm formation against *Klebsiella pneumoniae*. AgNP concentration of 100 *μ*g/ml was evaluated through the percentage biofilm inhibition 64% for *K. pneumoniae* strain MF953600 and 86% for MF953599 [41].

The results are consistent with the findings of a previous study by Shakibaie et al. [13], reporting that SeNPs at the concentration of (0–16 *μ*g mL^−1^) therapy inhibited the biofilm formation of *S. aureus*, *P. aeruginosa*, and *P. mirabilis* by 42, 34.3, and 53.4%, respectively. In their study, SeNPs was made with spherical shape and diameter range of 80 to 220 nm synthesized by *Bacillus* sp. [12]. Contrary to this study results, Haney demonstrated that iron oxide nanoparticles at a concentration of 0.2 mg/mL could increase biofilm biomass [42].

The results showed that the potential of SeNPs as an inhibitor of biofilm formation against *V. cholerae* was described (Figure 9).

In short, this study showed that SeNPs could be used as an antibiofilm and antibacterial agent against *V. cholerae* infections. The limitation of our research included the lack of an in vivo study, which is intended to be carried out in future efforts.

## 5. Conclusion

The synthesized nanostructures exhibited high antibiofilm and antibacterial activity against *V. cholerae*, which is considered as a major public health concern. In conclusion, SeNPs could be considered as a new therapeutic nanostructure with high antibacterial and antibiofilm potential and used as a promising alternative for *V. cholerae* infections therapy in clinical settings. Therefore, SeNP might be useful for various pharmaceutical applications.

## Figures and Tables

**Table 1:** Biofilm formation of *V. cholerae* O1 ATCC 14035 strain before and after the exposure to different concentrations of SeNPs.

Concentration SeNPs (*μ*g/ml)	Mean of OD ± SD	Biofilm formation	Formula
200	0.031 ± 0.01	Nonadherent	OD < ODc
100	0.059 ± 0.01	Nonadherent	OD < ODc
50	0.110 ± 0.05	Weak	ODc < OD < 2ODc
25	0.914 ± 0.12	Strong	OD < 4ODc
12.5	1.088 ± 0.10	Strong	OD < 4ODc
0	1.125 ± 0.09	Strong	OD < 4ODc
Control negative	0.064 ± 0.01	Nonadherent	ODc−
Control positive	1.28 ± 0.12	Strong	ODc+

**Table 2:** Antibacterial test of nanoparticle selenium against bacteria.

Reference	NP size (nm)	Tested bacteria	Antibacterial parameters
[9]	35.6 ± 7.5	*E. coli*	1, 5, 10 *μ*g/mL
		*S. aureus*	

[35]	8–20	*E. coli*	23 *μ*g/mL
		*S. aureus*	11.7 *μ*g/mL

[36]	30–50	*Enterococcus faecalis*	25 *μ*g/ml

[37]	80	*MRSA MDR*	8.6–4.2 *μ*g/mL
		*K. pneumonia*	26.2–0.4 *μ*g/mL

[14]	10–50	*E. coli*	25 *μ*g/ml
		*S. aureus*	

## Data Availability

The datasets used and/or analyzed during the current work are available from the corresponding authors on reasonable request.
